# Irisin levels in genetic and essential obesity: clues for a potential dual role

**DOI:** 10.1038/s41598-020-57855-5

**Published:** 2020-01-23

**Authors:** Stefania Mai, Graziano Grugni, Chiara Mele, Roberta Vietti, Luisella Vigna, Alessandro Sartorio, Gianluca Aimaretti, Massimo Scacchi, Paolo Marzullo

**Affiliations:** 1 Istituto Auxologico Italiano, IRCCS, Laboratory of Metabolic Research, Ospedale S. Giuseppe, via Cadorna 90, 28824 Piancavallo di Oggebbio, (VB) Italy; 2 Istituto Auxologico Italiano, IRCCS, Division of Auxology, Ospedale S. Giuseppe, via Cadorna 90, 28824 Piancavallo di Oggebbio, (VB) Italy; 3 Istituto Auxologico Italiano, IRCCS, Division of General Medicine, Ospedale S. Giuseppe, via Cadorna 90, 28824 Piancavallo di Oggebbio, (VB) Italy; 4 University of Piemonte Orientale, Department of Translational Medicine, via Solaroli 17, 28100 Novara, Italy; 5 Istituto Auxologico Italiano, IRCCS, Laboratory of Clinical Neurobiology, Ospedale S. Giuseppe, via Cadorna 90, 28824 Piancavallo di Oggebbio, (VB), Italy; 6 University of Milan, Department of Clinical Sciences and Community Health, via Commenda 19, 20122 Milan, Italy

**Keywords:** Homeostasis, Obesity

## Abstract

Irisin is conventionally regarded as a myokine involved in the browning of white adipose tissue, energy expenditure and glucose tolerance. Its potential link to fat accumulation and metabolic dysfunction is debated. We sought to explore the relationship between circulating irisin and components of body composition in two different phenotypes of severe obesity. For this purpose, 30 obese adults with Prader-Will syndrome (PWS) (age 35.7 ± 1.5 y, BMI 45.5 ± 1.5 kg/m^2^) and 30 adult controls with common obesity (age 34.9 ± 1.7 y, BMI 46.8 ± 1.4 kg/m^2^) underwent analysis of irisin levels, metabolic profile, body composition and resting energy expenditure (REE). Normal irisin levels were obtained from a group of 20 lean donors (age 32.4 ± 1.5 y, BMI 23.8 ± 0.8 kg/m^2^). Expected differences in body composition and metabolic profile existed between study groups. PWS exhibited lower muscle mass (p < 0.001), FFM (p < 0.001), REE (p < 0.001), as well as insulin (p < 0.05), HOMA-IR (p < 0.05) and triglycerides levels (p < 0.05) than controls with common obesity. In PWS, irisin levels were significantly lower and overall less dispersed than in controls with common obesity (p < 0.05), while being similar to values recorded in lean subjects. To explore the relation between irisin and body composition in obesity, univariate correlation analysis in the obese populations as a whole showed positive associations between irisin and muscle mass (p = 0.03) as well as REE (p = 0.01), which disappeared when controlled for the PWS status. Noticeably, a positive association became evident between irisin and %FM after controlling for the PWS status (p = 0.02). Also positive were associations between irisin and insulin (p = 0.02), HOMA-IR (p = 0.02) and triglycerides (p = 0.04). In stepwise multivariable regression analysis, irisin levels were independently predicted by the PWS status (p = 0.001), %FM (p = 0.004) and triglycerides (p = 0.008). Current results suggest that obese adults with PWS harbor lower irisin levels than individuals with common obesity. The divergent models of obesity herein studied suggest a potential link between circulating irisin and muscle mass and metabolic dysfunction relating to adiposity.

## Introduction

Prader-Willi syndrome (PWS) is a rare genetic disorder characterized by neonatal hypotonia associated with poor suck, followed by the lack of a sense of satiety with obsessive craving for food in childhood and gradual development of morbid obesity in adulthood unless eating is externally controlled^1–4^. Further clinical features include body dysmorphisms, developmental disability, cognitive and behavioral disorders, endocrine dysfunctions leading to short stature, hypogonadism and hypothyroidism^1^. PWS occurs because of the lack of expression of genes located on the paternal chromosome 15q11.2-q13. Three main genetic mechanisms have been recognized in determining PWS: deletion of the paternal chromosome 15 (del15q11-q13), maternal uniparental disomy of chromosome 15 (UPD15), and imprinting defects^2^.

The body composition of obese adults with PWS substantially differs from that of subjects with common obesity. Fat mass (FM) is generally higher in PWS and comparative analyses of fat compartments also revealed that obese PWS subjects predominantly accumulate subcutaneous adipose tissue (SAT), while visceral adipose tissue (VAT) is usually less represented compared to patients with common obesity^5–7^. Adding evidence to these phenotype differences, in PWS fat-free mass (FFM) is significantly lower and muscle function as well as resting energy expenditure (REE) are impaired compared to subjects with common obesity, although REE results unaltered when adjusted for FFM^3,4^. There is consolidated evidence that obese PWS patients harbor a more favorable metabolic profile as compared to individuals with common obesity, including lower insulin levels, higher insulin sensitivity, increased adiponectin concentrations, as well as milder degrees of systemic inflammation and liver steatosis^5–8^.

Irisin, first identified in 2012 as a muscle-derived factor capable of inducing the browning of white adipose tissue (WAT)^9^, acts as a myokine and increases energy expenditure and glucose tolerance^10^. Mice and humans studies investigating irisin-mediated pathways have demonstrated that exercise increases the expression of peroxisome proliferator-activated receptor (PPAR)-γ coactivator, (PGC)−1α, which results in the expression of fibronectin type III domain containing (FNDC)5, a transmembrane protein acting as the precursor of irisin, as confirmed by evidence that irisin is produced by proteolytic cleavage of FNCD5 at the level of cell membrane^9^. Once it is released into the circulation, irisin is able to stimulate the expression of the uncoupling protein-1(UCP1) and the browning of WAT, which prompts an increase in total body energy expenditure by increasing UCP1-mediated thermogenesis^9,11^.

On the other hand, there is accumulating evidence that FNDC5/irisin also acts as an adipokine, as it is both expressed and secreted by WAT in rats and humans^12^. In rodents, FNDC5/ irisin is primarily secreted from adipocytes of SAT and, in lower amount, from adipocytes of VAT^12^. Studies in normal-weight as well as obese and extremely obese subjects reported that circulating irisin is positively associated with BMI and body weight^13–17^. Consequently, irisin is associated with several measures of adiposity, such as FM, waist circumference, waist-to-hip ratio^14,15,17,18^, as well as muscle mass^13,15^. Less clear is the role played by irisin in glucose metabolism, and the association relating irisin levels to blood glucose and insulin levels or insulin resistance is currently debated^13,17,19,20^. Circulating irisin has been found to be associated with increased odds of harboring the metabolic syndrome and insulin resistance^21^, while an opposite association exists between insulin sensitivity and circulating irisin^22,23^.

Interest on circulating irisin in PWS is currently limited to a study on patients with average BMIs of 29.2 kg/m^2^, where an association between irisin and total and low-density lipoprotein(LDL)-cholesterol was described^24^. Intriguingly, this study observed higher levels of salivary irisin in PWS compared to normal-weight controls, while plasma irisin levels did not differ between the two populations^24^. Because the obese phenotype of adults with PWS markedly differs from that of BMI-matched subjects with common obesity, the present study was undertaken to explore circulating levels of irisin in two adult models of obesity in relation to body composition and metabolic profiling, so as to gain further insights on its role as a myokine or adipokine in the obese setting.

## Methods

### Patients

This study enrolled 60 patients, consisting of 30 PWS adults with obesity (11 M/19 F; age, 35.7 ± 1.5 years; BMI, 45.5 ± 1.5 kg/m^2^) and 30 BMI-matched control subjects with common obesity (11 M/19 F; age, 34.9 ± 1.7 years; BMI, 46.8 ± 1.4 kg/m^2^), referred to our institution for work-up and rehabilitation of obesity and its comorbidities. In addition to the previous, a control group constituted by 20 age-and gender-matched healthy normal-weight controls (9 M/11 F; age, 32.4 ± 1.5 years; BMI, 23.8 ± 0.8 kg/m^2^) were recruited among the Institution’s employees and included for the comparative analysis regarding circulating irisin levels. All PWS individuals received a diagnosis based on typical syndromic features confirmed by molecular genetic studies of chromosome 15, including 15q11-q13 deletion in 24 (11 males and 13 females) and UPD15 in the remaining 6 females. Exclusion criteria were: type 1 or type II diabetes mellitus, autoimmune and/or chronic inflammatory disorders, chronic obstructive pulmonary disease, history of neoplasms or degenerative diseases, previous chronic steroid treatment, kidney or cardiac disorders, uncontrolled hypothyroidism, exposure to glucocorticoids. With respect to hormone replacement, 11 PWS patients were treated with rhGH, 5 female PWS patients were receiving estrogens and 3 PWS patients were receiving levothyroxine treatment. No patient with common obesity was undergoing pharmacological therapies at the time of the study. The experimental procedure was approved by the ad hoc Ethical Research Committee of the Istituto Auxologico Italiano, Verbania, Italy. A written informed consent was obtained from the PWS patients and their parents or guardians, and from the obese and normal weight control participants. The study protocol conformed to the guidelines of the European Convention on Human Rights and Biomedicine concerning biomedical research.

### Body measurements

All subjects underwent body measurements wearing light underwear, in fasting conditions after voiding as described previously^25,26^. Weight and height were measured to the nearest 0.1 kg and 0.1 cm, respectively, using standard methods. BMI was expressed as body mass (kg)/height (m)^2^. Obesity was defined for any BMI over 30 kg/m^2^ ^2,27^. Waist circumference (WC) was measured midway between the lowest rib and the top of the iliac crest after gentle expiration; hip measurements were taken at the greatest circumference around the nates. FM and FFM were determined by bioelectrical impedance analysis (BIA model 101/S Akern, Florence, Italy) as described previously^26^. According to the vectorial analysis, patients with fluid overload were excluded to minimize errors in estimating FM and FFM. REE was expressed in kilocalories/24 h and determined in a thermoregulated room (22–24 °C) by computed open-circuit indirect calorimetry, measuring resting oxygen uptake and resting carbon dioxide production by a ventilated canopy (Sensormedics, Milan, Italy) at 1-min intervals for 30 min, expressed as 24 h value, as described previously^28^.

### Laboratory tests

Blood samples were drawn under fasting conditions, centrifuged, and stored at −80 °C until required. Serum irisin levels were assessed using a commercially available human ELISA kit EK-067–29 (Phoenix Pharmaceutics, Inc, Burlingame, CA, USA) in accordance with the manufacturer’s instructions. This ELISA is specific for human irisin, and quality controls were included in all ELISA measurements with the results falling within the expected range. All samples were analyzed in duplicate. Intra-assay and inter-assay coefficients of variation (CV) of irisin immunoassays were less than 10% and 15% respectively, and minimum detectable concentration was 1.5 ng/mL. Serum leptin concentrations were quantified using a commercially available ELISA kit (Mediagnost GmbH, Reutlingen, Germany) with overall inter- and intra-assay CVs of 6.8–8.3% and 5.5–6.9% respectively. Routine laboratory data included levels of C-reactive protein, aspartate aminotransferase (AST), alanine aminotransferase (ALT), gamma-glutamyl transpeptidase (GGT), glucose, total cholesterol, high-density (HDL), low-density lipoprotein (LDL) cholesterol and triglycerides (TG) measured by enzymatic methods (Roche Diagnostics, Mannheim, Germany). Levels of insulin were measured using a Cobas Integra 800 autoanalyzer (Roche Diagnostics, Indianapolis, IN, USA). Insulin resistance was calculated by the homeostatic model of insulin resistance (HOMA-IR) index: insulin (mIU/L) × [glucose (mmol/L)/22.5]^29^. A HOMA-IR value greater than 2.0 was considered indicative of insulin resistance, as obtained in a sample of the Italian population^30^.

### Western immunoblotting

Blood samples were drawn in fasting conditions, then they were centrifuged, separated, aliquoted, and kept at −80 °C until assays in single batches. Serum albumin was depleted using Qproteome Albumin/IgG depletion kit (Qiagen, Hilden, Germany). Recombinant irisin (human, aa 32–143) was purchased from Adipogen (AG-40B-0136, San Diego, USA). For Western immunoblots (WIB), all serum samples were size-fractionated on 15% Sodium Dodecyl sulphate polyacrylamide gel electrophoresis (SDS-PAGE) under reducing conditions and electro-transferred to immuno-blot polyvinylidene difluoride (PVDF) membranes (Millipore, Millerica, MA, USA). Next, membranes were blocked in 5% BSA in TBST (0.2% Tween 20 in Tris Buffered Saline) and then incubated with polyclonal rabbit anti-irisin antibody (1:1500, catalog G-067–17 from Phoenix Pharmaceutical, Burlingame CA, USA). Membranes were subsequently incubated for 1 hour with a specific secondary horseradish peroxidase (HRP)-conjugated antibody (Sigma Aldrich, Saint Louis, MO, USA). Immunoreactive proteins were detected using enhanced Femto enhanced chemiluminescence (Thermo Scientific, Rockford, USA) and images were captured using Azure Biosystems c300 (Azure Biosystems, CA, USA). The proteins were quantified by Pierce BCA Protein Assay Kit (Thermo Scientific, Rockford, USA) and equal loading was confirmed by Ponceau S staining (Sigma Aldrich, Saint Louis, MO, USA).

### Statistical analysis

Statistical analysis was performed using SPSS version 18 (Somers, NY, USA). Values are expressed as mean ± SEM. Comparative analyses between all three groups were performed by Kruskall-Wallis test with Dunn’s correction, and by ANOVA between males or female in each group. Spearman’s correlation analysis was used to identify significant associations between variables of interest. A stepwise multivariable regression analysis was used to evaluate the relationship between metabolic, anthropometric or biochemical parameters and variations in irisin levels. Three multilinear models were built which included the obese phenotype (common obesity = 0; PWS = 1) in association with parameters of body composition (model 1: muscle mass, FFM and %FM), metabolic homeostasis (model 2: insulin, HOMA-IR, triglycerides and leptin) or a combination of the significant predictors from the previous two models (model 3: %FM and triglycerides). β coefficients and significance values obtained from the regression models are reported. A p value < 0.05 was considered as statistically significant.

## Results

Anthropometric and biochemical characteristics of the three study groups are summarized in Tables 1 and 2 respectively. In the obese populations, BMI values ranged between 31.5–62.1 kg/m^2^ and were comparable between the two obese groups. Expected differences in adiposity and related measures existed between lean controls and the obese groups, while muscle mass was similar between PWS and normal weight group. When PWS patients were compared to controls with common obesity, they showed lower values of muscle mass (p < 0.001), FFM (p < 0.001) and REE (p < 0.001), while the REE/FFM ratio did not differ between the two groups. Likewise, no difference in FM was documented due to the extremely obese phenotype of these populations. Analysis of metabolic parameters showed lower insulin (p < 0.05), HOMA-IR (p < 0.05), triglycerides levels (p < 0.05) and liver transaminases (AST, and ALT p < 0.05 for both) in PWS than controls with common obesity, suggestive of a healthier metabolic profile in the former.

**Table 1:** Summary of anthropometric data obtained in normal weight control subject (NW), PWS subjects (PWS) and controls with common obesity (OB).

Variables	NW (n = 20)	PWS (n = 30)	OB (n = 30)
Males/females	9/11	11/19	11/19
Age (years)	32.4 ± 1.5	35.7 ± 1.5	34.9 ± 1.7
BMI (kg/m^2^)	23.8 ± 0.8	45.5 ± 1.5^b^	46.8 ± 1.4^b^
Weight (kg)	64.3 ± 3.11	103.7 ± 3.7^b,d^	127.1 ± 3.8^b^
Height (cm)	163.7 ± 1.0	151.1 ± 1.5^b,d^	165.0 ± 1.7
Waist (cm)	81 ± 2.7	122.2 ± 2.6^b^	129.5 ± 2.7^b^
Hip (cm)	101 ± 1.7	131.1 ± 2.6^b^	136.8 ± 2.7^b^
Waist-to-hip ratio	0.80 ± 0.02	0.93 ± 0.02^b^	0.90 ± 0.02^b^
FM (%)	25.2 ± 1.7	50.4 ± 1.1^b^	49.6 ± 1.3^b^
FFM (kg)	50.9 ± 2.6	50.7 ± 1.7^d^	62.5 ± 2.4^a^
Muscle mass (kg)	28.1 ± 1.6	26.2 ± 0.9^d^	35.8 ± 1.5^b^
REE (kcal/day)	1637.5 ± 59.5	1542.6 ± 50.3^d^	2049.6 ± 78.4^a^
REE/FFM	32.5 ± 1.2	30.7 ± 0.7	33.0 ± 0.7

Data are expressed as mean ± SEM. Comparative analyses between all three groups were performed by Kruskall-Wallis test with Dunn’s correction.

^a^P < 0.05, NW vs PWS or OB; ^b^P < 0.001, NW vs PWS or OB; ^c^P < 0.05, PW vs OB; ^d^P < 0.001, PW vs OB.

For abbreviations: BMI, body mass index; FM, fat mass; FFM, fat free mass; REE, resting energy expenditure.

**Table 2:** Summary of biochemical data obtained in normal weight control subject (NW), PWS subjects (PWS) and controls with common obesity (OB).

Variables	NW (n = 20)	PWS (n = 30)	OB (n = 30)
Irisin (ng/mL)	16.7 ± 1.4	18.1 ± 1.1^c^	25.3 ± 1.6^a^
Leptin (ng/ml)	9.7 ± 2.4	69.4 ± 8.0^b^	54.0 ± 5.3^b^
Fasting glucose (mg/dL)	91.6 ± 2.6	95.7 ± 5.0	91.2 ± 1.8
Insulin (mIU/L)	8.6 ± 1.1	10.2 ± 0.9^c^	14.8 ± 1.3^a^
C-Peptide (μg/L)	na	2.1 ± 0.1	3.5 ± 0.4^d^
HOMA-IR	2 ± 0.3	2.4 ± 0.3^c^	3.4 ± 0.3^a^
AST (U/L)	18.3 ± 3.3	18.0 ± 1.1^c^	24.1 ± 2.0
ALT (U/L)	22.2 ± 2.1	22.6 ± 2.7^c^	36.2 ± 4.3^a^
GGT (U/L)	19.5 ± 5.2	29.7 ± 7.7	29.8 ± 3.7
TG (mg/dL)	65.6 ± 5.9	90.7 ± 5.7^a,c^	118.4 ± 7.5^b^
Tot CHO (mg/dL)	183.3 ± 9.3	173.1 ± 7.3	177.5 ± 5.9
LDL CHO (mg/dL)	115.5 ± 7.5	112.8 ± 37.0	111.1 ± 27.4
HDL CHO (mg/dL)	64.3 ± 4.7	50.3 ± 2.2^b^	46.5 ± 2.1^b^

Data are expressed as mean ± SEM. Comparative analyses between all three groups were performed by Kruskall-Wallis test with Dunn’s correction.

^a^P < 0.05, NW vs PWS or OB; ^b^P < 0.001, NW vs PWS or OB; ^c^P < 0.05, PW vs OB; ^d^P < 0.001, PW vs OB.

For abbreviations: AST, aspartate aminotransferase; ALT, alanine aminotransferase; GGT, gammaglutamyl transpeptidase; tot CHO, total cholesterol; LDL CHO, low density lipoprotein cholesterol; HDL CHO, high density lipoprotein cholesterol; TG, triglycerides.

na = not applicable.

**Figure 1:**
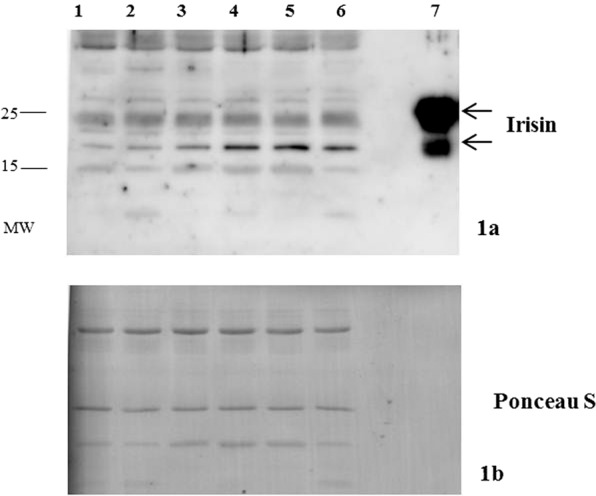
Representative cropped Western immunoblot of irisin (**1a**) expression in sera from three patients with PWS (lines 1–3) and three controls with common obesity (lines 4–6). Recombinant irisin is represented in line 7. Equal loading was confirmed by Ponceau S staining on the same gel (**1b**). Full-length blots are presented in Supplementary Fig. 1. MW = molecular weight.

**Figure 2:**
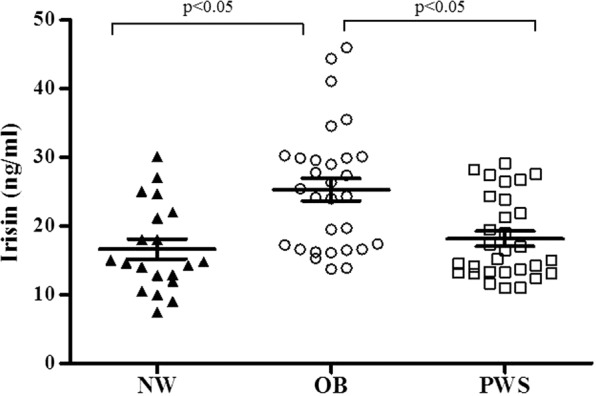
Individual values and mean ± SEM of circulating irisin levels obtained in normal-weight controls (NW, triangle), controls with common obesity (OB, circle) and PWS patients (PWS, square). Lines represent mean and SEM values in the populations.

Analysis of irisin expression in sera representative of the two obese populations was initially verified by WIB, to clarify potential controversies on irisin detection in human serum^31^. The WIB of sera from three PWS patients and three controls with common obesity detected two bands at approximately 25 and 22 kDa that was consistent with that of recombinant irisin, which was thus adequately detected by the commercial antibody used herein (Fig. 1). The intensity of the 22 kDa band corresponding to irisin appeared to be weaker in PWS than controls with common obesity. The ELISA study showed that irisin concentrations were lower and less variable in obese PWS subjects that controls with common obesity (Fig. 2) and the exclusion of 3 outliers from the group with common obesity (irisin concentrations, >40 ng/ml), left the difference between PWS and controls with common obesity unchanged (mean values 23.2 ± 1.2 ng/ml, p < 0.05). Alternatively, irisin levels were comparable between PWS patients and lean controls and were, therefore, lower in lean controls than subjects with common obesity. In gender-based analysis, irisin levels did not differ between males and females in PWS (17.9 ± 1.8 and 18.3 ± 1.4 ng/ml) as well as controls with common obesity (26.1 ± 2.4 and 24.8 ± 2.1 ng/ml), while males from the lean group exhibited non-significantly higher irisin levels than females (20.7 ± 3.1 and 12.7 ± 1.8 ng/ml, p = 0.06).

**Figure 3:**
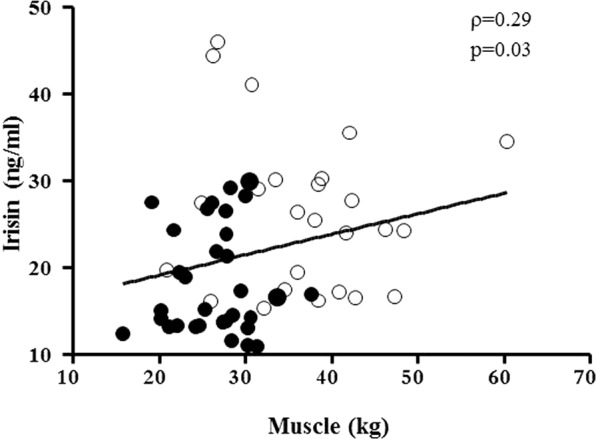
Relationship between irisin and muscle mass in the PWS subjects and controls with common obesity as a whole. Closed circles, obese PWS subjects; open circles: controls with common obesity.

To analyze irisin behavior in relation to components of body composition in obesity, a correlation analysis was originally performed in separate obese groups, but results were not statistically significant likely due to the small sample size of each group. To add statistical power and weigh the potential role of covariates, datasets from both obese groups were merged (Table 3). Irisin levels were positively associated with muscle mass (Fig. 3, Table 3) and this correlation was maintained after omitting 3 outliers in irisin levels (r = 0.35, p = 0.007). However, this positive association was lost after controlling for the PWS status, suggesting that variations in irisin levels likely reflected differences in muscle mass between groups. In unadjusted analysis, there was no correlation between irisin and %FM (Table 3), while this association became significantly positive after controlling for the PWS status (r = 0.30, p = 0.02). This circumstance seems to suggest an overall influence of FM on circulating irisin once the PWS status is accounted for. A positive correlation was also observed between irisin and REE, which was maintained after controlling for age, gender and BMI (r = 0.26, p < 0.05), but disappeared when controlling for the PWS status. This observation likely confirms the role of PWS and the divergent anthropometric phenotype on REE. In stepwise multivariable regression, irisin did not predict REE, while FFM (β = 0.65, p < 0.0001) and muscle mass did (β = 0.27, p = 0.007).

**Table 3:** Spearman’s correlation analysis between irisin levels and anthropometric and biochemical parameters in the PWS subjects and controls with common obesity as a whole.

Parameters	Irisin levels	
	**ρ**	**P**
Age (years)	0.020	0.9
PWS status	**−0.46**	**0.0001**
BMI (kg/m^2^)	0.10	0.5
FM (%)	0.064	0.6
FFM (kg)	0.23	0.08
Muscle mass (kg)	**0.29**	**0.03**
REE (kcal/day)	**0.32**	**0.01**
REE/FFM	0.098	0.4
Leptin (ng/ml)	−0.018	0.9
Glucose (mg/dl)	−0.093	0.5
Insulin (mIU/L)	**0.29**	**0.02**
HOMA-IR	**0.31**	**0.02**
C- Peptide (μg/L)	**0.28**	**0.03**
TG (mg/dL)	**0.25**	**0.04**
Tot CHO (mg/dL)	0.079	0.5
HDL CHO (mg/dL)	−0.020	0.9
LDL CHO (mg/dL)	0.12	0.4

For PWS status: PWS = 1, common obese = 0. Significance is shown in bold characters.

For abbreviations: BMI, body mass index; FM, fat mass; FFM, fat free mass; REE, resting energy expenditure; tot CHO, total cholesterol; LDL CHO, low density lipoprotein cholesterol; HDL CHO, high density lipoprotein cholesterol; TG, triglycerides.

Analysis of metabolic parameters showed a positive correlation between irisin and triglycerides, which remained unaltered after controlling for the PWS status (r = 0.27, p < 0.05). When glucose homeostasis was taken into account, irisin was positively associated with insulin and C-peptide levels as well as with HOMA-IR. The correlation between irisin and insulin (r = 0.30, p < 0.05) or C-peptide (r = 0.30, p < 0.05) persisted after controlling for age, sex and BMI, while it disappeared when the PWS status was accounted for.

Based on the previous results, a number of stepwise multivariable regression models were performed to assess the predictive effect of anthropometric and metabolic parameters on circulating irisin (Table 4). In the first two models, PWS status emerged with %FM and triglycerides as significant predictors among the phenotypic and metabolic covariates of irisin levels. When significant predictors were combined, PWS status acted as the strongest predictor of circulating irisin, followed by %FM and triglycerides levels.

**Table 4:** Regression coefficients derived from the stepwise multivariable regression analysis conducted in the PWS subjects and obese controls as a whole on irisin as the dependent variable.

Multivariable regression analysis	Included variables			Excluded variables
		β	P value	
**Model 1**	Group	−0.51	0.0001	Muscle mass, FFM
	FM (%)	0.28	0.02	
**Model 2**	Group	−0.31	0.02	HOMA-IR, Insulin, leptin
	TG (mg/dL)	0.28	0.03	
**Model 3**	Group	−0.42	0.001	none
	FM (%)	0.34	0.004	
	TG (mg/dL)	0.32	0.008	

Group (PWS = 1, obese controls = 0) was introduced as independent variable in all models. Other independent variables introduced in the three models: model 1: %FM, muscle mass, FFM; model 2: insulin, HOMA-IR, triglycerides, leptin; model 3 (combined from the previous): %FM and triglycerides. β standardized coefficients and p values are shown.

For abbreviations: HOMA-IR, homeostatic model of insulin resistance; FM, fat mass; FFM, fat free mass; TG, triglycerides.

## Discussion

The present study analyzed circulating irisin levels in young obese adults with PWS and common obesity in relation to body compartments and metabolic profile. Results showed that PWS patients harbor lower irisin levels than obese controls, and that significant associations seem to relate irisin levels to muscle mass, REE, insulin resistance and triglycerides levels. In this obese setting, the strongest independent predictors of irisin levels were PWS status, %FM and triglycerides.

PWS is an example of genetic human obesity related to hypothalamic dysfunction, with several interacting endocrine and metabolic abnormalities that have previously been thought to influence body composition^32,33^. PWS patients have a blunted tendency to accumulate visceral adiposity and generally show better cardiometabolic profiles compared to common obesity, despite being exposed to an enhanced risk of premature life-threatening complications^34^. The recently discovered myokine irisin has attracted attention for its potential role in adipose tissue accumulation and ability to induce the browning of WAT. According to this view, irisin increases the mitochondrial content and UCP-1 concentrations in WAT, thereby increasing its thermogenic activity^9^. In humans, circulating irisin is also positively associated with parameters of adiposity such as BMI^13,16–18^. It has been consequently proposed as a promising therapeutic target for obesity and metabolic disorders^19,35^. Our study initially focused on verifying irisin immunoreactivity in sera due to a non-canonical start codon in humans^36^. Western immunoblot with commercially available antibodies was able to detect irisin in sera from PWS and obese controls at a molecular weight of approximately 22 and 25 kDa, which likely resulted from dimerization and/or glycosylation^11,15,37,38^. Following this preliminary result, a comparative investigation using a reliable ELISA kit^39^ allowed us to observe that obese PWS patients had lower irisin levels than controls with common obesity, while being similar between PWS and lean subjects. Also, circulating irisin appeared less variable in PWS and lean donors than in subjects with common obesity, a finding that substantiates and expands to the PWS status previous observations obtained in obese and lean subjects^15^. Our results obtained in sera are somewhat similar to those reported in a study by Hirsch *et al*., who showed comparable irisin levels in plasma between overweight PWS patients and a group on normal-weight controls, while documenting higher irisin levels in PWS when measured in saliva^24^. We are inclined to interpret the underlying divergences with caution, because differences in sample number, anthropometric characteristics, BMI and biological matrices could play a role.

However, a number of clues make our results consistent with the recognized metabolic roles of irisin, and will be followingly discussed. First, under normal metabolic conditions muscle mass is acknowledged as the main source of circulating irisin^9–11^. Previous work from Huh and coworkers showed that circulating irisin declines in parallel with age-related muscle loss, and documented a strong positive association between irisin and biceps circumference, implying that muscle mass is the main determinant of circulating irisin levels in humans^13^. Other studies prompted similar evidence of a positive correlation between irisin and fat-free mass^15,18,40,41^. Hence, the positive correlation observed between irisin and muscle mass agrees with the divergent obese phenotypes of the populations studied^42^. It should be noted that the correlation analysis was only conducted in the obese populations as a whole after excluding lean controls, so as to determine the specific effect of PWS and excluding the potential statistical interference relating to the lean group. Previous work showed that obese PWS subjects harbor lower muscle mass and FFM than BMI-matched obese individuals without PWS, while being similar to FFM values observed in lean controls^5,43^. Moreover, individuals with common obesity are tipically engaged in stronger spontaneous workout than PWS, who exhibit muscle hypotonia and poor attitude to exercise^42^. Hence, the loss of significance in the correlation observed in our study between irisin and muscle mass when corrected for PWS status likely reflects the divergent phenotype of our obese populations. Furthermore, irisin is a myokine involved in exercise, energy expenditure and thermogenesis^9,44^, and is associated with REE in conditions of extreme BMI^16^. Although our results showed a positive correlation between irisin and REE, this association disappeared after controlling for the PWS state, suggesting the influence of PWS on these variables. Together, our results suggest that irisin activities as a myokine can be influenced by genetic differences in the obese phenotype.

Secondly, fat mass has been suggested to act as a potential determinant of circulating irisin^13,16^. In mice, it has been calculated that approximately 28% of circulating irisin originates from adipose tissue^9^. This contribution has been proposed to switch from relative to predominant in the case of WAT excess and/or metabolic WAT dysfunction associated with VAT accumulation^31^. Roca Rivada *et al*. hypothesized that pathological conditions like obesity make adipose tissue and metabolic dysfunction more relevant for irisin production than other body tissues^12^. This circumstance could imply that the relative contribution of muscle or adipose tissue to irisin production varies depending on the physiological setting, adipose tissue dysfunction and fat/lean mass proportion^31^. If this holds true, the association between irisin and %FM seen here pinpoints a potential role for irisin as an adipokine, possibly related to the different fat partitioning between the two obese populations.

Thirdly, there is evidence that irisin may act as a metabolic gauge^14,21,45,46^, and the positive association seen between irisin and triglycerides agrees with this inference. It is worth mentioning that adipose tissue from obese subjects shows an upregulation of lipolysis^47^, and the high level of fatty acids, through the portal vein, could flow directly into the liver. This could promote hypertriglyceridemia as a result of triglycerides synthesis^48^. Although this and other findings could imply that circulating irisin simply reflects lipid homeostasis in obesity, irisin may represent in condition of metabolic dysfunction an adaptive or even compensatory response directed from the skeletal muscle to the endocrine pancreas to signal insulin resistance^49^. Thus, not only irisin intervenes to regulate glucose homeostasis and insulin sensitivity^39,50^, but its concentrations may also reflect the consequence of a metabolic burden^49^. In line with these evidences, we observed a positive correlation between irisin and insulin, C-peptide and HOMA-IR, but these associations were dependent on the PWS status. Positive associations between irisin and insulin resistance were previously reported in non-diabetic adults^20,21^ as well as in conditions of extreme BMI, such as obesity and anorexia nervosa, after adjusting for group-dependent covariates^16^. Opposite findings were reported in a recent study in Caucasian subjects with different degrees of obesity^19^. Whether this circumstance depends on the diverging phenotype or genetic makeup of our obese groups remains to be clarified.

Our study has some limitations. Analysis of body composition by BIA does not allow accurate measurement of body components, particularly under conditions of severely altered BMI^51^. Although dual X-ray absorptiometry (DXA) is considered the gold standard for the assessment of body composition, the very obese phenotype of the patients herein studied exceeded the DXA weigh limit in many cases. Nevertheless, BIA has already proven useful in clinical settings investigating irisin in relation to body composition^15,40,41,46^. Because our study was conducted in PWS patients with severe obesity, its results may not extend to non-obese PWS patients. Finally, the cross-sectional nature of our study does not allow to draw conclusions on the potential response of irisin to exercise and/or weight loss in condition of PWS. Notwithstanding these limitations, this study has important strengths, including the inclusion of a consistent sample of adult PWS patients as well as the evaluation of multiple anthropometric and biochemical parameters.

Together, our findings suggest that circulating irisin levels increase in comon obesity but not in PWS-related obesity, and this discrepancy likely reflects differences both in fat and muscle mass between these obese populations. The potential influence of a genetic component associated with PWS cannot be, however, entirely excluded. More likely, this divergence highlights the potential dual role of irisin as a myokine and adipokine, which remains to be clarified in longitudinal studies.

## Supplementary information

Supplementary Information.
