# Supplementary material for: Irisin levels in genetic and essential obesity: clues for a potential dual role
Source: Sci Rep. 2020 Jan 23;10:1020. doi: 10.1038/s41598-020-57855-5 (PMC6978420; doi:10.1038/s41598-020-57855-5)
Supplement: Supplementary file 1 — Supplementary Information. [file 41598_2020_57855_MOESM1_ESM.docx]

**Irisin levels in genetic and essential obesity: clues for a potential dual role**

Stefania Mai^1*^, Graziano Grugni^2^, Chiara Mele ^3,5^, Roberta Vietti^1^, Luisella Vigna^4^, Alessandro Sartorio^2^, Gianluca Aimaretti^5^, Massimo Scacchi^3^, Paolo Marzullo^3,5^

**Affiliations**

^1^Istituto Auxologico Italiano, IRCCS, Laboratory of Metabolic Research, Ospedale S. Giuseppe, via Cadorna 90, 28824, Piancavallo di Oggebbio (VB), Italy

^2^Istituto Auxologico Italiano, IRCCS, Division of Auxology, Ospedale S. Giuseppe, via Cadorna 90, 28824, Piancavallo di Oggebbio (VB), Italy

^3^Istituto Auxologico Italiano, IRCCS, Division of General Medicine, Ospedale S. Giuseppe, via Cadorna 90 28824, Piancavallo di Oggebbio (VB), Italy

^4^Istituto Auxologico Italiano, IRCCS, Laboratory of Clinical Neurobiology, Ospedale S. Giuseppe, via Cadorna 90, 28824, Piancavallo di Oggebbio (VB), Italy

^5^University of Piemonte Orientale, Department of Translational Medicine, via Solaroli 17, 28100, Novara, Italy.

**Keywords:** irisin, obesity, Prader-Willi syndrome, muscle mass, fat-free mass, fat mass

***Corresponding author:**

Stefania Mai

Istituto Auxologico Italiano, IRCCS

Laboratory of Metabolic Research, Ospedale S. Giuseppe

Via Cadorna 90, 28824

Piancavallo di Oggebbio (VB), Italy

s.mai@auxologico.it

**Supplementary Figure 1:** Representative Western immunoblot of irisin (1a) expression in sera from three patients with PWS (lines 1-3) and three controls with common obesity (lines 4-6). Recombinant irisin is represented in line 7. Equal loading was confirmed by Ponceau S staining on the same gel (1b) . The figure shows the uncropped full length western blots of gels presented in Figure 1 of the main article. MW= molecular weight.


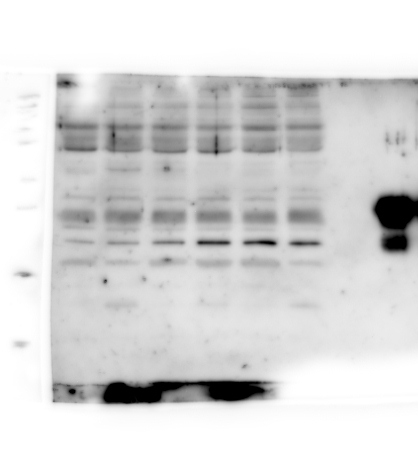


15

25

**1 2 3 4 5 6 7**

**1 2 3 4 5 6 7**

**1a**

**Irisin**

10

35

55

70


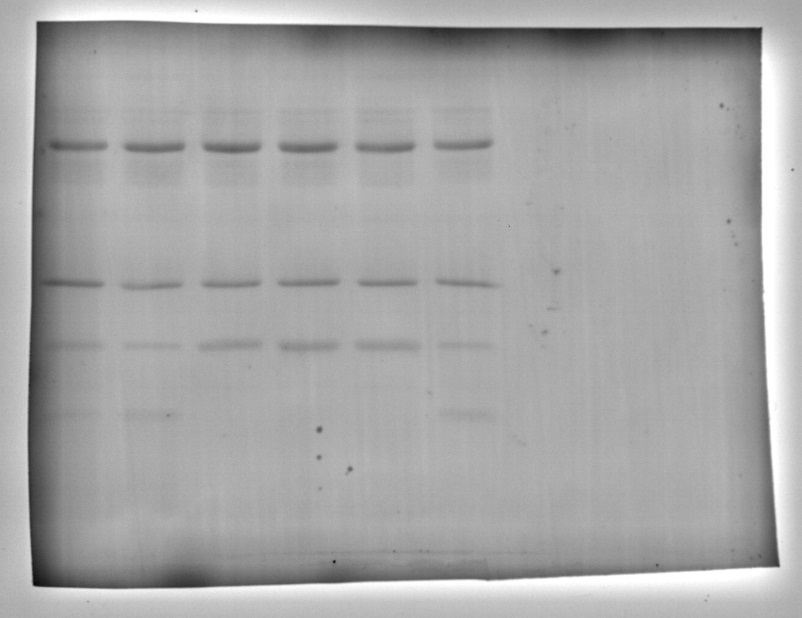


**1b**

**Ponceau S Staining**
